# Locally relevant, ethically urgent: Defending SASOP’s stance on transgender and non-binary youth

**DOI:** 10.4102/sajpsychiatry.v32i0.2543

**Published:** 2026-01-23

**Authors:** KL Dunkle, Ingrid Lynch, Sakhile Msweli, Ronald Addinall-van Straaten, Pierre Brouard, Jenna-Lee de Beer-Procter, Nkanyiso Madlala, Chris McLachlan, Madeleine Muller, Simon Pickstone-Taylor, Mershen Pillay, Ariane Spitaels, Anastacia Tomson, Elma de Vries

**Affiliations:** 1 Centre for Sexualities, AIDS and Gender, University of Pretoria, Pretoria, South Africa; 2 Critical Studies in Sexualities and Reproduction, Rhodes University, Makhanda, South Africa; 3 KwaZulu-Natal Department of Health, Durban, South Africa; 4 Department of Social Work and Social Development, University of Cape Town, Cape Town, South Africa; 5 Centre for Human Rights, University of Pretoria, Pretoria, South Africa; 6 Department of Psychology, Stellenbosch University, Cape Town, South Africa; 7 Centre for Public Mental Health, University of Cape Town, Cape Town, South Africa; 8 Department of Psychology, Faculty of Social Sciences, University of South Africa, Pretoria, South Africa; 9 Department of Family Medicine and Rural Health, Faculty of Health Sciences, Walter Sisulu University, East London, South Africa; 10 Department of Family Medicine, Cecilia Makiwane Hospital, East London, South Africa; 11 Department of Psychiatry, Faculty of Health Sciences, University of Cape Town, Cape Town, South Africa; 12 Disciplines of Audiology and Speech-language Pathology, School of Health Sciences, University of KwaZulu-Natal, Durban, South Africa; 13 Speech and Language Therapy Programme, Institute of Education, Massey University, Auckland, New Zealand; 14 Department of Paediatrics and Child Health, University of Cape Town, Cape Town, South Africa; 15 Red Cross Children’s Hospital, Cape Town, South Africa; 16 My Family GP, Cape Town, South Africa; 17 Shemah Koleinu, Cape Town, South Africa; 18 School of Medicine, Faculty of Health Sciences, Nelson Mandela University, Gqeberha, South Africa

## Introduction

In the Global North, access to gender-affirming care for transgender and non-binary youth is increasingly undermined by politically motivated attacks that ignore clinical evidence and patient rights.^1^ In this climate, the 2024 position statement by the South African Society of Psychiatrists (SASOP) on the care of transgender and non-binary youth^2^ represents an ethically sound, evidence-informed, and culturally attuned framework that places patient dignity, legal rights, and clinical best practices at its core. The recent scientific letter in this journal from Donkin et al.^3^ risks undermining this progress by repeating a selectively sceptical and ideologically biased narrative originating from the Global North that fails to reflect either the global scientific consensus or South African constitutional values. This letter defends SASOP’s current position by examining the flaws in Donkin et al.’s critique and source material and affirming the methodological rigour and ethical clarity of recently published guidelines for care of transgender and non-binary youth.^4,5^

## Bias and methodological limitations of *The Cass Review* and other sources

Donkin et al.’s letter centres much of its case on the findings of *The Cass Review*, a report on gender identity services for children and young people in England commissioned by National Health Service (NHS) England and NHS Improvement, whose content primarily comprises policy recommendations specific to the NHS.^6^ *The Cass Review* has been widely criticised for methodological opacity, a lack of stakeholder accountability, and the exclusion of affirming outcome data.^7,8,9^ Although *The Cass Review* acknowledges the importance of ‘research evidence, clinical expertise and patient values’,^6^ it fails to fully meet these criteria. The lead author had no expertise in the treatment of or research into gender dysphoria in children and adolescents, and individuals with lived experience of being transgender had limited input into the process.^7,9^ This contravenes best practices for evidence synthesis and guideline development, which call for appropriate subject-matter expertise and meaningful accountability to stakeholders to mitigate bias and strengthen validity.^10^ By analogy, a review of cisgender women’s health that overtly excluded clinicians or researchers specialising in the field and rebuffed input from cisgender women themselves would rightly be regarded as biased, uninformed and fundamentally flawed.

Moreover, much of *The Cass Review’s* framing and conclusions align closely with talking points advanced by US- and UK-based groups that actively oppose gender-affirming care.^8^ This ideological alignment extends to other key sources cited by Donkin et al. In addition to directly citing the misleadingly named Society for Evidence-Based Gender Medicine (SEGM) – a group described by a Yale School of Medicine review as spreading ‘biased and unscientific content’ to restrict access to gender-affirming care^11^ – five of the citations underpinning Donkin et al.’s arguments are authored by individuals affiliated with SEGM and Genspect.^i^ Both organisations are documented anti-LGBTQ+ groups^12^ and identified as central to what the Southern Poverty Law Center describes as the ‘anti-LGBTQ+ pseudoscience network’.^13^ One of these five sources, the ‘WPATH Files’, attempts to discredit the World Professional Association for Transgender Health (WPATH) through selective and misleading excerpting of posts from internal professional forums.^14^ These sources do not constitute a neutral or empirically grounded critique, but rather reflect a coordinated output of a tightly networked, agenda-driven ecosystem.^15^ This pervasive ideological backdrop raises serious concerns about the applicability of Donkin et al.’s claims within a South African rights-based clinical context.

## Global consensus confirms gender-affirming care is effective and necessary

Donkin et al. criticise the SASOP position statement for supporting clinical guidelines issued by WPATH^16^ and the South African HIV Clinicians’ Society (SAHCS)^17^ (Table 1). They take particular issue with the South African guidelines’ legal alignment with the *Children’s Act*^18^ (which provides that children over 12 who demonstrate sufficient maturity can consent to medical treatment), while disregarding the guidelines’ emphasis on the importance of parent or caregiver involvement.

**TABLE 1:** Overview of recent youth gender-affirming care guidelines and policy statements.

Aspect	South African guidelines		Recent international clinical guidelines			Context-specific policy report
	SAHCS GAHC (2021)	PsySSA (2025)	WPATH SOC8 (2022)	French SFEDP (2024)	German AWMF (2025)	Cass Review, NHS England (2024)
Ideal Mental Healthcare Provider Involvement	Encouraged for psychosocial support and supporting informed consent to medical intervention (pp. 38–45).	Central role for affirming mental health practices (pp. 19–23).	Required for assessment and ongoing support, with flexibility based on regional context and available resources (pp. S50–51, S56, S71–75).	Mandatory for evaluation, consent support, and family involvement (pp. 2–6).	Integral; all decisions made in multidisciplinary settings (pp. 206, 207).	Recommends holistic mental health screening to inform individualised care plans (Rec 2–3, pp. 29–31).
Social Transition for Youth	Supported, ideally with caregivers ‘engagement and support from therapist as needed (pp. 21–22, 42–43).	Affirmed as valid identity development (pp. 31, 53).	Supported when carried out carefully, considering benefits and risks (pp. S75–S78).	N/A (endocrine guideline).	Supported, to be shaped according to the needs of the child (pp. 60, 71–72, 120).	Recommends early access to clinical professionals for families considering social transition of pre-pubertal children; acknowledges that social transition often precedes clinical presentation (Rec 4, pp. 30–32).
Recommended Role of Parents or Caregivers	Supports caregivers ‘involvement and notes that a supportive family system leads to better patient mental health outcomes. Emphasises the role of providers in educating and supporting caregivers (pp. 21–23, 40–45).	Recommended inclusion for youth; supports family systems approaches (pp. 57–60).	Recommended unless involvement is harmful or abusive (pp. S57–59, S69, S73).	Required for youth under 16; involvement is key to support (pp. 6, 8, 13).	Expected for all minors; legal guardians part of care discussions with shared decision-making (pp. 72, 91, 129, 146–148, 174–176, 201–202).	Supports caregiver involvement and provision of evidence-based guidance to both caregivers and patients; recommends mental health support for caregivers and siblings as appropriate (Rec 3, p. 31).
Informed Consent for Medical Interventions	Informed consent required; includes assessment of maturity and ability to consent; every effort should be made to involve caregivers in decisions as this leads to better outcomes (pp. 22–23). Notes that the SA *Children’s Act* supports consent to medical care from age 12.	N/A (mental health guideline).	Informed consent required; includes assessment of maturity and ability to consent; caregiver involvement when safe. No fixed age threshold because of international variations in context and law (pp. S57–59, S116).	Informed consent is required, with assessment of maturity and ability to consent; caregiver involvement expected for < 16 (pp. 2–3).	Informed consent required, with no fixed age threshold. Decisions based on individual maturity, distress, and persisting ICD-11 gender incongruence. Emphasises assessed capacity, documentation, and shared decision-making (pp. 152, 174, 200–201, 218).	Consent must be informed and developmentally appropriate. While 16 is the local legal threshold for medical consent, the review recommends delaying gender-affirming hormonal care until 18 (Rec 8 and Ch 16, pp. 35, 192–197).
Puberty Blockers (GnRHa)	Can be prescribed from Tanner Stage 2+; requires psychosocial assessment and involvement of a paediatric endocrinologist (pp. 21–23, 43–45, 57–58).	N/A (mental health guideline).	Recommended Tanner Stage 2+, multidisciplinary assessment (pp. S112–114).	Recommended case-by-case; requires ICD-11 dysphoria, Tanner Stage 2+ (pp. 3–4).	Recommended Tanner Stage 2+, presence of ICD-11 dysphoria, multidisciplinary assessment (pp. 182, 188–190, 194–195).	Recommends use only in research protocols (pp. 32–35).
Fertility Counselling	Strongly recommended, particularly prior to gender-affirming hormone therapy (GAHT) or surgery (p. 58).	N/A (mental health guideline).	Recommended prior to GAHT or surgery (pp. S63, S75, S118–119).	Recommended prior to GAHT or surgery (p. 8).	Recommended prior to GAHT or surgery (p. 219).	Recommended prior to GAHT or surgery (Rec 10, p. 35).
Gender-Affirming Hormone Therapy (GAHT)	Supported for mature adolescents with informed consent as above, with multidisciplinary team review (pp. 23–24, 57–58).	N/A (mental health guideline).	Supported with informed consent as above, with multidisciplinary team review (pp. S114–116).	Supported with informed consent as above, with multidisciplinary team review (pp. 4–8).	Supported with informed consent as above, with multidisciplinary review (pp. 206, 209–210).	Recommends GAHT from age 16 only with a strong clinical rationale; prefers delaying until 18. Advocates pairing with national multidisciplinary team review and fertility counselling (Rec 7–10, p. 35).
Desistance, Detransition or Retransition	Not specifically discussed, but if there is a change of mind at any point regarding transition, it is supported (pp. 21–22).	Mentioned as a highly individual experience that does not invalidate a person’s earlier gender journey (pp. 87–88).	Recognised but warns against overemphasis; stresses support for individual pathways (pp. S41–S42).	Recognised but warns against overemphasis (p. 2).	Desistance and retransition acknowledged; integrated into care planning (pp. 25, 55–59, 105, 130, 163, 189, 223, 234–237).	Recognised and recommends that appropriate care be available (Rec 25). Notes that ‘[y]oung people may also choose to stop hormone treatment but carry on identifying as transgender or non-binary’ (p. 188).
Pathologisation of Gender Diverse Identities (e.g. Use of ‘ROGD’ or ‘Social Contagion’ Framings)	Not explicitly mentioned; multiple identity pathways affirmed.	Not explicitly mentioned; guidelines reject pathologising frameworks (pp. 20, 68).	Rejects ‘ROGD’ and ‘social contagion’ framings (p. S45).	Not mentioned.	Rejects ‘ROGD’ and ‘social contagion’ framings (p. 100).	Critiques the term ‘social contagion’ as inadequate, oversimplifying and potentially distressing. Highlights complex and individual biopsychosocial model for aetiology (pp. 117, 121–122).

ROGD, rapid-onset gender dysphoria; PsySSA, Psychological Society of South Africa; WPATH, World Professional Association for Transgender Health; SAHCS, South African HIV Clinicians Society; SFEDP, French Society of Pediatric Endocrinology and Diabetology; AMWF, Association of Scientific Medical Societies; NHS, National Health Service.

Donkin et al. also fail to engage with recent guidelines from the French Society of Pediatric Endocrinology and Diabetology (SFEDP)^4^ and the German Association of Scientific Medical Societies (AWMF)._5_ Both were developed through robust, multidisciplinary and transparent processes. These guidelines conclude that gender-affirming care for adolescents, when provided with comprehensive psychosocial support and informed consent, is effective and appropriate. They explicitly reject the discredited notions of ‘rapid-onset gender dysphoria’ (ROGD) and ‘social contagion’, both invoked in Donkin et al.’s critique.^19,20,21^ Instead, they advocate for evidence-based, individualised pathways that acknowledge the fluidity of gender development while centring the well-being of youth.^4,5^

Donkin et al.’s letter complains that no explicit mention is made in these guidelines of ‘exploratory therapy’, an undefined process with no established long-term outcome data and informed by a focus on seeking traumatic or pathological roots for gender identity rather than treating it as a neutral outcome.^22^ They likewise fail to acknowledge that contemporary guidelines for gender-affirming care (Table 1) recognise that desistance and retransition occur and are valid outcomes within a gender-affirming framework. These trajectories are supported without pathologisation or coercion, underscoring the role of clinicians as facilitators of care rather than gatekeepers of identity. By contrast, Donkin et al. focus narrowly on the small minority of patients who have detransitioned with regret, thereby erasing the experiences of the overwhelming majority of transgender youth who benefit from access to care or experience harm and distress when care is denied or made inaccessible.^23,24^

## Ethical and professional responsibilities call for context-sensitive, affirming care

Focusing solely on narratives of regret, while ignoring affirming experiences and the harms resulting from denied care, is ethically indefensible. Such a narrow lens distorts the clinical landscape, violates principles of evidence-based medicine, and disregards the human rights of patients. The Psychological Society of South Africa’s (PsySSA) updated 2025 guidelines^25^ explicitly call on providers to confront their own prejudices and to uphold a non-pathologising, affirming stance. Importantly, these guidelines were authored by a diverse group of psychology professionals grounded in South Africa’s constitutional principles, and they emphasise the clinician’s duty to provide compassionate, respectful and context-sensitive care.^25^

This context includes South Africa’s rich cultural heritage. Many precolonial societies understood gender as fluid, socially situated, and relational – embedded in roles, responsibilities, and community recognition rather than fixed identity categories.^26^ Colonial and missionary legal systems suppressed these relational understandings, imposing rigid binaries and sex-at-birth classifications that still constrain how gender diversity is socially and legally navigated today.^27^ In contemporary South Africa, some communities continue to hold beliefs that ancestors can manifest in descendants of a different gender. While such views are neither universal nor uncontested, they offer some gender-diverse South Africans meaningful cultural frameworks for recognition. For example, a person assigned female at birth may be recognised as *mkhulu* [grandfather], or someone assigned male as *gogo* [grandmother].^28^ Failing to engage with these heterogeneous and historically grounded expressions of gender reflects an implicitly colonial framing. Opening space to challenge rigid gender binaries – and to facilitate social, legal and yes, medical transition – creates room to reclaim expansive and contextually grounded understandings of gender, including decolonial ones.

Crucially, objections such as those raised by Donkin et al. obscure the actual crisis: the widespread lack of access to gender-affirming healthcare for transgender and non-binary youth in South Africa. Gender-affirming care has been available in South Africa since the 1970s, yet services remain overstretched, under-resourced, and inaccessible to most – especially economically marginalised youth and those outside major urban centres.^29,30^ While some argue about the pace or appropriateness of care, the far more urgent problem is that most transgender youth are unable to access any care at all. Many avoid health services altogether because of adversarial and/or overtly abusive behaviour from providers.^29^ Ideologically compromised calls to delay or withdraw services do nothing to address this reality, and in fact risk deepening health inequality.^30^

## The South African Society of Psychiatrists’ position statement is clinically rigorous, legally sound and ethically urgent

The 2024 South African Position Statement on Evidence-Based Care for Transgender and Gender-Diverse Young People – endorsed by a wide coalition of 32 medical, legal and rights organisations, plus over 150 individual clinicians and scholars – affirms the need for accessible, evidence-based and non-discriminatory care, as well as the need to deconstruct rigid gender binaries.^31^ The South African Society of Psychiatrists’ position statement is thus not an outlier. It is rooted in South African jurisprudence and ethical thought, which values the necessity of holistic care and the bodily autonomy and right to self-determination of the child.

In addition to this ethical and legal grounding, SASOP’s endorsement of gender-affirming care is supported by data. International longitudinal studies, although imperfect, demonstrate that access to puberty blockers and gender-affirming hormones can reduce rates of depression, anxiety and suicidality among youth.^32^ *The Cass Review*,^6^ by contrast, dismisses many of these findings on narrow methodological grounds while failing to propose feasible alternatives. The WPATH,^16^ SAHCS,^17^ AWMF^5^ and SFEDP^4^ recognise that perfect data are unattainable in paediatric populations and advocate for the provision of gender-affirming care to adolescents.

Against this backdrop, the claim that SASOP’s position is driven by ‘activism’ rather than science is misguided. The statement was developed by child and adolescent psychiatrists drawing on a robust body of evidence and clinical guidelines.^2^ It reflects the prevailing consensus among leading medical and mental health professionals working in the field of transgender health. At the same time, this dismissal by terming SASOP’s position as activism overlooks the historic role of advocacy in medical progress. Health professionals have long played a critical role in advocating for ethical care in their fields, including the depathologisation of homosexuality, the destigmatisation of HIV and AIDS, and the expansion of mental health services. The South African Society of Psychiatrists’ recognition of the rights of gender-diverse youth continues this tradition of evidence-based professional advocacy. The position statement is far from politically captured; it is scientifically sound and ethically urgent.

Finally, the assertion that youth are incapable of informed consent underestimates the capacity of adolescents, contradicts South African law, and infantilises youth who are navigating their identities. Similar claims have historically been used to deny adolescents’ access to abortion care – arguments ultimately rejected in favour of upholding bodily autonomy and health rights.^33^ South Africa’s *Children’s Act* affirms that minors deemed competent may consent to medical treatment. The South African Society of Psychiatrists’ position statement aligns with this legal standard while emphasising the role of caregivers and mental health professionals in facilitating informed decisions.

In conclusion, SASOP’s position statement on the care of transgender and non-binary youth is clinically rigorous, legally sound and contextually rooted. The statement’s emphasis on psychosocial support, staged interventions and informed consent reflects global best practices – not ideological extremism. Donkin et al.’s critique relies on discredited sources produced by well-documented Global North anti-LGBTQ+ groups, which are out of step with clinical consensus and have been superseded by robust evidence syntheses. It offers no viable alternative beyond delaying or withholding care – approaches that are not neutral but constitute a form of clinical neglect for young people requiring timely, gender-affirming support.

We urge psychiatric professionals to stand firmly in support of an evidence-based, human rights-driven future for transgender youth care in South Africa. This requires structural support and systemic alignment: embedding gender-affirming care into clinical training across levels, expanding rural access through decentralised models, and explicitly including gender-affirming care alongside other key areas such as mental health, sexual and reproductive health, and social support in national adolescent strategies. Finally, this work should meaningfully partner with trans-led organisations in service design and delivery – its raison d’être is to serve, not invalidate, transgender youth.

## Footnotes

i Citation affiliations include E. Abbruzzese (SEGM co-founder); H. Barnes and S. Baxendale (keynote speakers at SEGM NYC, 2023); M. Hughes (director of Genspect Canada; her paper is published by Environmental Progress, founded by Genspect advisor M. Shellenberger); and a paper reporting SEGM funding for publishing fees with the lead author (S.C. Jorgensen) disclosing a SEGM-funded travel grant.
