# Corrigendum: Locally relevant, ethically urgent: Defending SASOP’s stance on transgender and non-binary youth

**DOI:** 10.4102/sajpsychiatry.v32i0.2679

**Published:** 2026-05-11

**Authors:** KL Dunkle, Ingrid Lynch, Sakhile Msweli, Ronald Addinall-van Straaten, Pierre Brouard, Jenna-Lee De Beer-Procter, Nkanyiso Madlala, Chris McLachlan, Madeleine Muller, Simon Pickstone-Taylor, Mershen Pillay, Ariane Spitaels, Anastacia Tomson, Elma de Vries

**Affiliations:** 1 Centre for Sexualities, AIDS and Gender, University of Pretoria, Pretoria, South Africa; 2 Critical Studies in Sexualities and Reproduction, Rhodes University, Makhanda, South Africa; 3 KwaZulu-Natal Department of Health, Durban, South Africa; 4 Department of Social Work and Social Development, University of Cape Town, Cape Town, South Africa; 5 Centre for Human Rights, University of Pretoria, Pretoria, South Africa; 6 Department of Psychology, Stellenbosch University, Cape Town, South Africa; 7 Centre for Public Mental Health, University of Cape Town, Cape Town, South Africa; 8 Department of Psychology, Faculty of Social Sciences, University of South Africa, Pretoria, South Africa; 9 Department of Family Medicine and Rural Health, Faculty of Health Sciences, Walter Sisulu University, East London, South Africa; 10 Department of Family Medicine, Cecilia Makiwane Hospital, East London, South Africa; 11 Department of Psychiatry, Faculty of Health Sciences, University of Cape Town, Cape Town, South Africa; 12 Speech and Language Therapy Programme, Institute of Education, Massey University, Auckland, New Zealand; 13 Disciplines of Audiology and Speech-language Pathology, School of Health Sciences, University of KwaZulu-Natal, Durban, South Africa; 14 Department of Paediatrics and Child Health, University of Cape Town, Cape Town, South Africa; 15 Red Cross Children’s Hospital, Cape Town, South Africa; 16 My Family GP, Cape Town, South Africa; 17 Shemah Koleinu, Cape Town, South Africa; 18 School of Medicine, Faculty of Health Sciences, Nelson Mandela University, Gqeberha, South Africa

In the published article, Dunkle KL, Lynch I, Msweli S, et al. Locally relevant, ethically urgent: Defending SASOP’s stance on transgender and non-binary youth. S Afr J Psychiat. 2026;32(0), a2543. https://doi.org/10.4102/sajpsychiatry.v32i0.2543, there was an error in the affiliation order of author 11, Mershen Pillay.

Instead of:

^12^Disciplines of Audiology and Speech-language Pathology, School of Health Sciences, University of KwaZulu-Natal, Durban, South Africa; and

^13^Speech and Language Therapy Programme, Institute of Education, Massey University, Auckland, New Zealand.

It should be:

^12^Speech and Language Therapy Programme, Institute of Education, Massey University, Auckland, New Zealand; and,

^13^Disciplines of Audiology and Speech-language Pathology, School of Health Sciences, University of KwaZulu-Natal, Durban, South Africa

The authors apologise for this error. The correction does not change the study’s findings of significance or overall interpretation of the study’s results or the scientific conclusions of the article in any way.

